# Antituberculosis Therapy-Induced Acute Liver Failure in a Renal Transplant Recipient: A Case Report

**DOI:** 10.7759/cureus.76263

**Published:** 2024-12-23

**Authors:** Selena Gajić, Ana Bontić, Aleksandra Kezić

**Affiliations:** 1 Nephrology, University Clinical Center of Serbia, Belgrade, SRB

**Keywords:** acute liver failure (alf), drug-induced acute liver failure, drug-induced hepatotoxicity (dih), renal transplant recipient, : tuberculosis

## Abstract

To prevent organ rejection, renal transplant (RT) recipients must take immunosuppressive medicines, which make them more susceptible to infections such as tuberculosis (TB). Hepatotoxicity, which can vary from asymptomatic increased liver enzymes to severe liver failure, is the most prevalent side effect of first-line antituberculosis (AT) drugs. Treating TB in RT patients involves unique concerns since AT medications might interact with immunosuppressive medications, potentially reducing efficacy or increasing toxicity. A 65-year-old RT recipient was diagnosed with active pulmonary TB 18 years after renal transplantation. He had drug-induced acute liver failure after initiating AT therapy, but his liver function improved after discontinuing AT medications and receiving supportive care.

## Introduction

To prevent organ rejection, renal transplant (RT) recipients must take immunosuppressive drugs for the rest of their lives. However, these treatments suppress the immune system and increase the RT recipient's risk of infections, including tuberculosis (TB), which can be acquired from external sources or reactivated from a latent state [1]. So, active TB disease is much more prevalent among RT recipients than in the general population, causing significant morbidity and mortality [2].

Although approximately 85% of TB cases are effectively cured, treatment-related side effects cause significant morbidity, resulting in diminished therapy effectiveness. Hepatotoxicity, which represents the damaging effect of certain drugs on liver cells, which can impair liver function, is the most common side effect, causing treatment discontinuation in 11% of patients treated with isoniazid (H), rifampicin (R), and pyrazinamide (Z) combination [3]. 

Drug-induced liver injury (DILI) presents as a spectrum of disease that can range from asymptomatic elevation of transaminase to acute liver failure, and it is a significant concern during TB treatment, so monitoring and adjusting medications are essential for minimizing this risk [4].

Treating TB in RT recipients presents unique challenges because commonly used AT drugs can interact with immunosuppressive drugs, potentially diminishing their effectiveness or increasing toxicity. The risk of liver damage from AT drugs, particularly H, R, and Z, further complicates treatment, as liver function is crucial for metabolizing both TB and immunosuppressive medications. Because of the underlying immunosuppression and the complex drug-drug interactions imposed by immunosuppressive medicines required to maintain the transplanted organ, TB disease in RT recipients is more difficult to manage [5].

This report presents the case of a 65-year-old RT recipient who developed drug-induced liver failure following AT therapy.

## Case presentation

The patient, a 65-year-old RT recipient, initially presented with weakness, cough, night sweats, and a 6-kg weight loss over two months. 

His past medical history included a cadaveric kidney transplantation (KT) due to end-stage renal disease of unknown origin, hypertension, aortic valve stenosis, and mitral valve insufficiency. His immunosuppressive maintenance therapy 18 years after the renal transplantation included cyclosporine (CyA) 0.7 g twice daily, mycophenolate mofetil (MMF) 750 mg twice daily, and prednisone (Pred) 7.5 mg daily. He smoked for 30 years, but he denied using alcohol and illicit drugs. 

On physical examination at the first admission, he was afebrile. His blood pressure was 110/60 mmHg. His heart rate was 97 bpm, with a precordial systolic murmur grade of 4/6. His respiratory rate was 15 per minute, with a vesicular breath sound on lung auscultation. His oxygen saturation in room air was 93%. On abdominal palpation, he had no tumors or organomegaly, and he had no peripheral edema.The laboratory results obtained at the time of the first admission are shown in Table 1. 

**Table 1:** Laboratory results at the first admission, at the second admission, and 20 days after discontinuation of antituberculosis (AT) drugs.

Test	Results at the first admission	Results at the second admission	Results 20 days after discontinuation of AT drugs	Normal range
White blood cell count	7.5 x 10^9^/L	17.3 x 10^9^/L	8 x 10^9^/L	3.4-9.7 x 10^9^/L
Hemoglobin	102 g/L	103 g/L	98 g/L	122-157 g/L
Platelets	166x10^9^ /L	372 x 10^9^ /L	135 x 10^9^/L	150-450 x 10^9^/L
Glucose	4.2 mmol/L	1.9 mmol/L	3.3 mmol/L	3.9-6.1 mmol/L
Urea	16.2 mmol/L	24.8 mmol/L	17 mmol/L	2.5-7.5 mmol/L
Creatinine	329 µmol/L	402 µmol/L	168 µmol/L	45-84 µmol/L
Sodium	136 mmol/L	136 mmol/L	136 mmol/L	135-148 mmol/L
Potassium	4.8 mmol/L	7 mmol/L	3.5 mmol/L	3.5-5.1 mmol/L
Total bilirubin	4.6 µmol/L	51 µmol/L	112 µmol/L	0.0-20.5 µmol/L
Conjugated bilirubin	-	39.4 µmol/L	77 µmol/L	0.0-20.5 µmol/L
Total protein	65 g/L	58 g/L	57 g/L	62-81 g/L
Aspartate transaminase	32 U/L	6377 U/L	84 U/L	0.0-37 U/L
Alanine transaminase	38 U/L	588 U/L	53 U/L	0.0-41 U/L
Alkaline phosphatase	85 U/L	253 U/L	176 U/L	40-120 U/L
Gamma glutamyltranspeptidase	40 IU/L	150 IU/L	258 U/L	0.0-55 U/L
Lactate dehydrogenase	423 U/L	4392 U/L	402 U/L	220-460 U/L
C-reactive protein	137 mg/L	116 mg/L	55 mg/L	0.0-5.0 mg/L
International normalised ratio	0.9	3.5	1.04	0.8-1.2
Activated partial thromboplastin time	26 s	53 s	30.5 s	22.0-32.0 s

**Figure 1:**
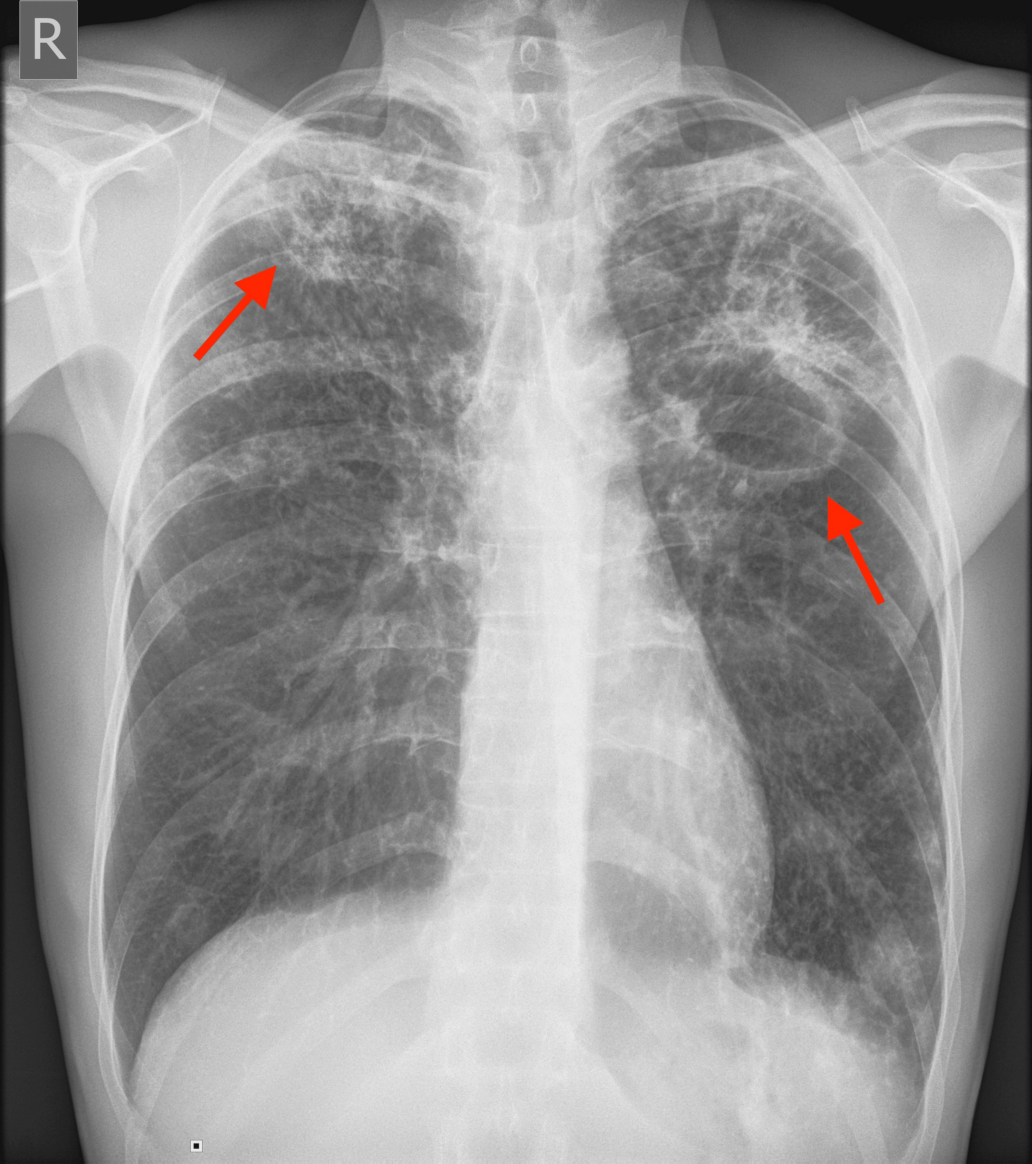
Anterior-posterior view chest X-ray showing bilateral cavernous changes (arrows).

A chest X-ray revealed bilateral cavernous changes typical for TB (Figure 1).

**Figure 2:**
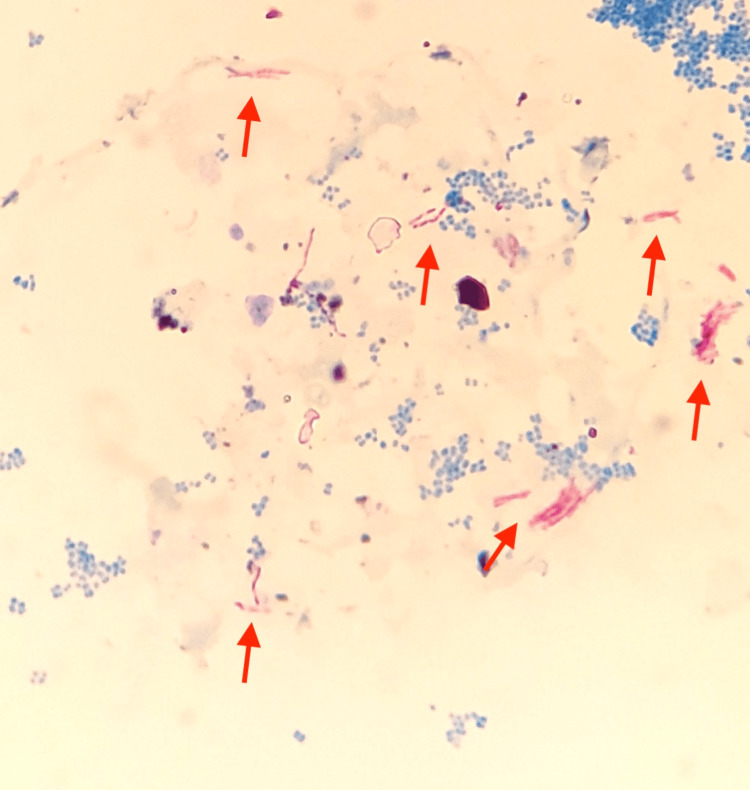
Positive acid-fast bacilli (AFB) staining from sputum under a light microscope (arrows).

A sputum sample was acid-fast bacilli (AFB) smear-positive (Figure 2).

**Figure 3:**
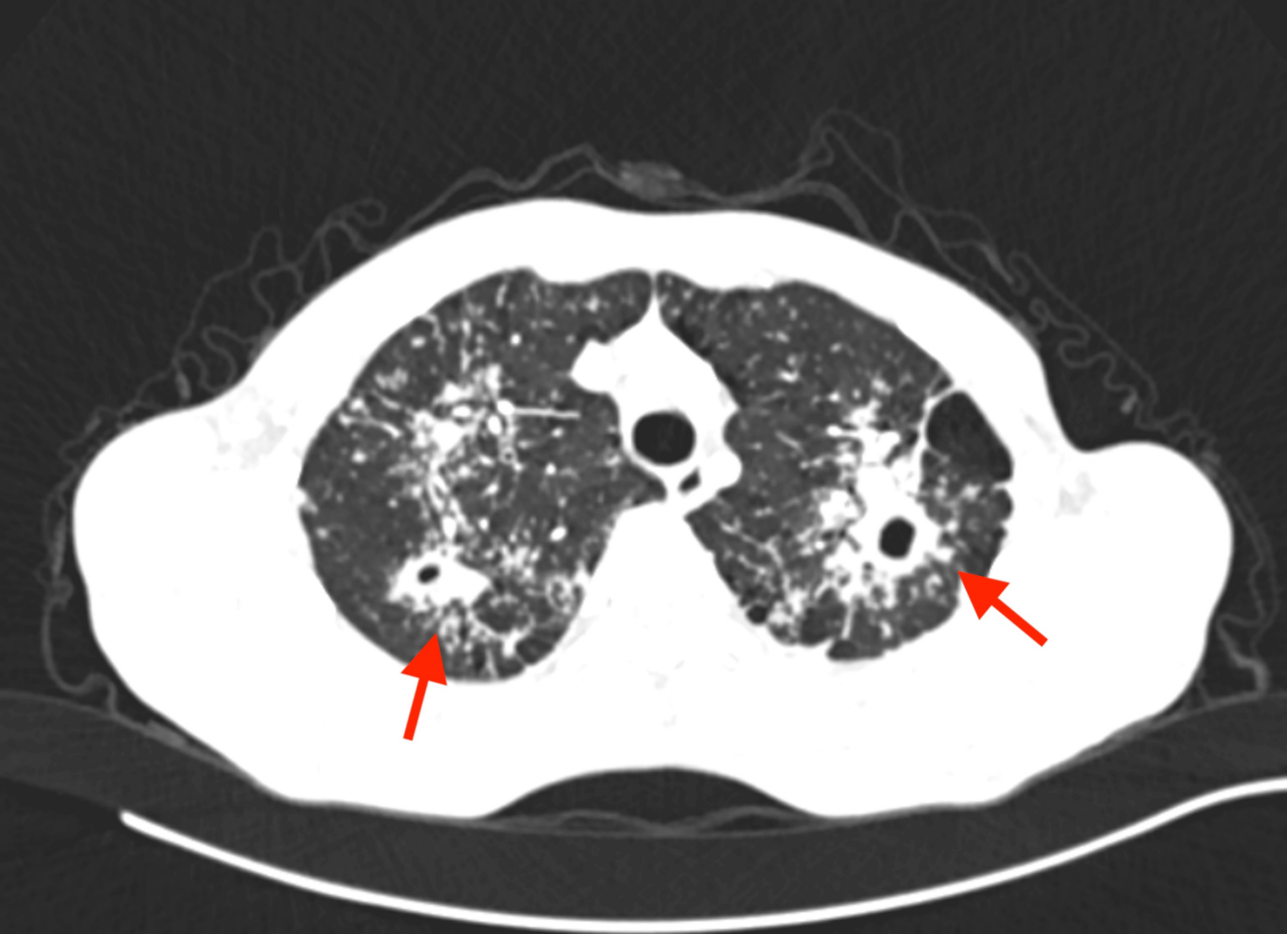
Chest CT scan showing caverns in both upper lobes (arrows).

Chest computed tomography (CT) scan showed caverns in both upper lobes, Nelson's segment, diffuse tree-in-bud opacification, a nodular soft tissue change on the left pleura, and enlarged hilar glands typical for TB (Figure 3).

The cartridge-based nucleic acid amplification test (NAAT) for rapid tuberculosis diagnosis and rapid antibiotic sensitivity test (Xpert MTB/RIF assay) was positive and did not detect resistance to R.

These symptoms, combined with imaging, NAAT, and sputum findings, led to a diagnosis of pulmonary TB.

The AT medication consisting of H 300 mg daily, R 600 mg daily, Z 800 mg daily, and ethambutol (E) 800 mg daily with pyridoxine 50 mg daily was initiated. The immunosuppressive medication was modified, and the calcineurin inhibitor (CNI), CyA, was replaced with a mammalian target of rapamycin (mTOR) inhibitor, everolimus 0.75 mg twice daily, MMF was reduced to 250 mg twice daily, and Pred was increased to 10 mg daily.

After seven days, he was released from the hospital with laboratory results of normal liver function. 

Seventeen days after starting AT therapy, he was readmitted with symptoms of nausea, decreased appetite, and vomiting. Clinical signs observed at readmission included somnolence and jaundice, with laboratory results showing a marked increase in liver enzymes (aspartate transaminase (AST) 6377 U/L, alanine transaminase (ALT) 588 U/L), elevated bilirubin (total bilirubin 51 µmol/L), and coagulation abnormalities (INR 3.5).

The laboratory results obtained at the time of the second admission are shown in Table 1.

**Figure 4:**
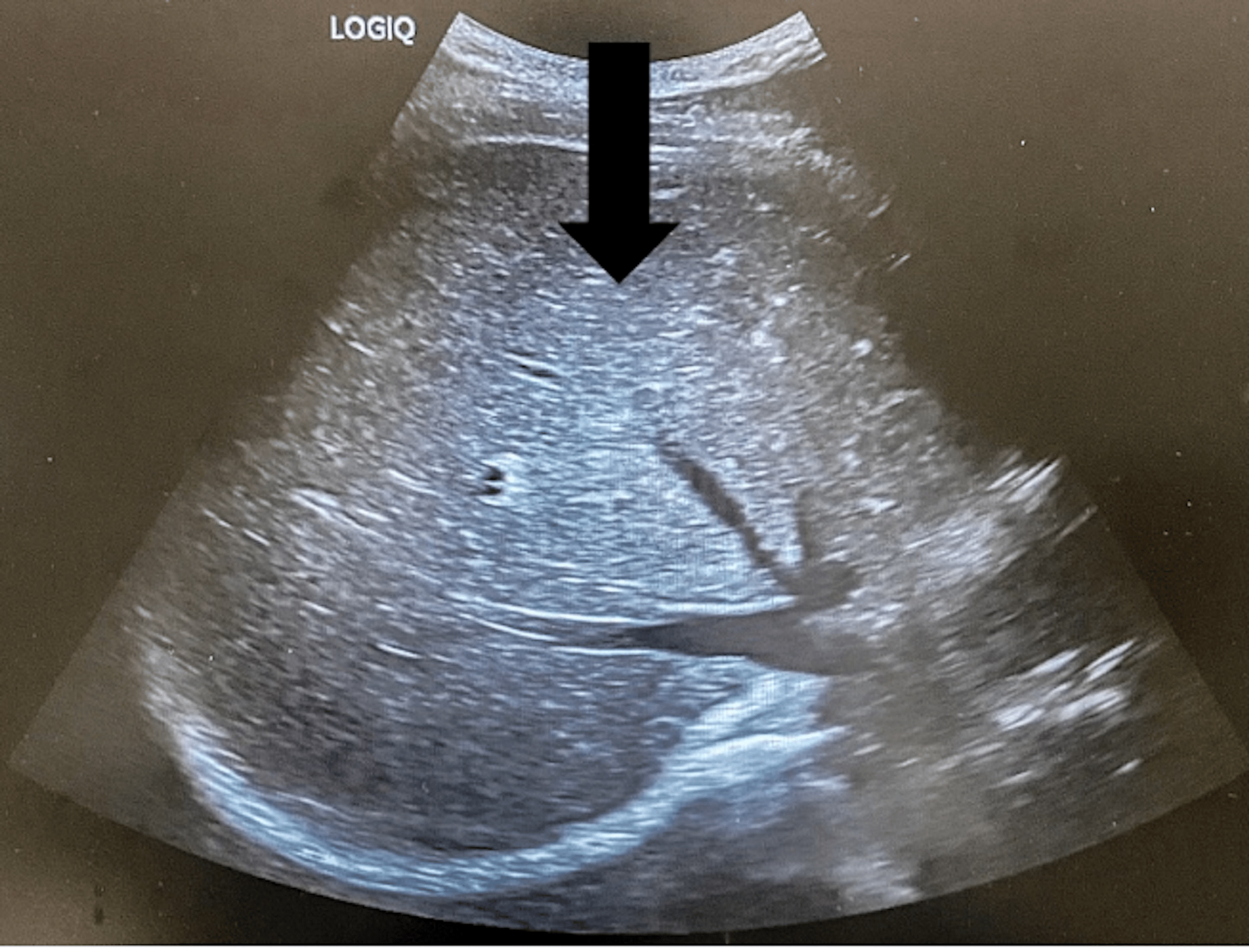
The abdominal cavity ultrasonography showing a hyperechogenic liver of average size (arrow).

Hepatitis A, B, and C virus tests were negative. Antinuclear antibodies, antimitochondrial antibodies, and antibodies to smooth muscles were negative. The abdominal cavity ultrasonography showed a hyperechogenic liver of average size. The bile ducts had not been dilated. There were no signs of gallstones on the gallbladder (Figure 4). 

Such findings were indicative of acute liver failure, likely induced by the AT drugs, prompting immediate discontinuation of the hepatotoxic AT drugs, and the patient's multidisciplinary treatment continued. 

Aspartate transaminase, alanine transaminase, and coagulation studies improved 20 days after discontinuing AT medications and continuing supportive care, but alkaline phosphatase, gamma glutamyltranspeptidase, and bilirubin remained elevated (Table 1). Because the initial AT protocol was hepatotoxic, the pulmonologist continued the treatment with alternative AT therapy, which included moxifloxacin 400 mg daily, E 800 mg daily, and streptomycin 1 g daily.

During treatment, massive rectorrhagia developed unexpectedly despite improvements in the patient's overall condition and laboratory tests of liver function. Pathohistological analysis of the resected colon with terminal ileum revealed acute perforative ulcerative diverticulitis. The dehiscence of the abdominal surgery wound worsened the patient's condition. We isolated *Pseudomonas aeruginosa *from a surgical wound swab. Antibiotic therapy was continued per the antibiogram, with daily wound dressing. Further treatment was complicated by sepsis, with a fatal outcome in the intensive care unit.

## Discussion

Compared to dialysis in end-stage renal disease, kidney transplantation (KT) significantly improves life expectancy and quality of life. However, the use of immunosuppressive medications, which are required to prevent RT loss, is directly linked to an increase in the incidence of infections and malignancies, which are one of the leading causes of morbidity and mortality in organ transplant recipients [6]. 

Active TB is more prevalent in RT patients than in the general population, ranging from 0.3% to 15.2% in endemic regions [7]. The majority of cases (51%) are present in the pulmonary form [8].

Active TB following KT might be caused by the reactivation of latent infection in the recipient or donor tissue or by a new infection after transplantation, of which the most prevalent is endogenous reactivation following KT [7]. Our patient was excluded for latent TB before transplantation. He had no history of known TB exposure and no recent travel history to endemic regions. 

Diagnosis of TB in RT patients is frequently difficult because the signs and symptoms in transplant recipients frequently differ from those in immunocompetent hosts. In transplant recipients, the traditional triad of fever, nocturnal sweats, and weight loss may occur less frequently. On imaging, the characteristic pulmonary cavitary lesions are frequently missing in posttransplant patients [9]. On admission, our patient was afebrile, but he had a history of other typical symptoms, as well as a chest X-ray and CT scan that revealed bilateral cavitary lesions, indicating TB.

Although the first-line drugs for the treatment of active TB in RT recipients are the same as for immunocompetent patients, including H, R, Z, and E, drug-drug interactions between AT and immunosuppressive drugs are frequent, making treatment more complex and challenging in RT patients [9-11]. Except for E, all first-line AT medications can produce hepatotoxicity, with P being the most potent [12]. Hepatotoxicity symptoms may range from asymptomatic elevated liver enzymes to severe liver failure [3].

Drug-induced hepatotoxicity is defined as an elevation of transaminases up to five times the upper limit of normal (ULN) in the absence of symptoms and up to three times ULN or twice ULN of bilirubin in the presence of symptoms, provided that other liver diseases, autoimmune hepatitis, and acute viral hepatitis are ruled out [12]. The median time between starting the AT medicine and developing clinical symptoms is 16 weeks [3]. 

Because our patient's liver function test results were in the reference range upon admission, the pulmonologist began TB treatment with a quadruple AT regimen of H 300 mg daily, R 600 mg daily, Z 800 mg daily, and E 800 mg daily. Due to the severe infection, the nephrologist modified the immunosuppressive therapy to everolimus 0.75 mg twice daily, MMF 250 mg twice daily, and Pred 10 mg daily. Laboratory liver function tests and everolimus levels were monitored on the third day. Despite a multidisciplinary approach, our patient developed drug-induced hepatotoxicity with allograft dysfunction 17 days after starting AT therapy. He had somnolence, nausea, decreased appetite, vomiting, and jaundice, as well as transaminases more than five times the ULN, bilirubin more than two times the ULN, and elevated international normalized ratio (INR). These findings were suggestive of acute liver failure, most likely caused by the AT medications.

All AT hepatotoxic first-line medicines should be discontinued once symptomatic hepatitis is detected. Clinical and biochemical improvements occurred immediately after discontinuing the causative medication in mild hepatotoxicity. According to American and British Thoracic societies' recommendations, once liver function is normal, first-line AT therapy can be reinitiated consecutively, starting with the safest drugs [12,13]. If hepatotoxicity recurs after first-line treatment, discontinue the medications and change to second-line therapy [12]. In our case, 20 days after discontinuing AT medications and obtaining supportive care, liver function partially improved, confirming the hepatotoxic effect of the initial AT regimen.

Following hepatogram stabilization, the pulmonary treatment continued with alternating AT therapy rather than first-line therapy, which included moxifloxacin, E, and streptomycin due to significant liver injury and the patient's poor general condition. The renal transplant and liver function remained stable throughout this treatment.

## Conclusions

Complications encountered during this case included: drug-induced liver failure following AT therapy, surgical wound infection with *Pseudomonas aeruginosa, *and sepsis in the intensive care setting. Ultimately, these complications led to a fatal outcome, despite discontinuation of the initial AT drugs and supportive care.

This case underscores the potentially life-threatening complications of AT therapy-induced hepatotoxicity and infection in immunocompromised organ transplant recipients, emphasizing the significance of a cautious medication selection and multidisciplinary approach involving close coordination between pulmonologists, hepatologists, nephrologists, and infectious disease specialists to balance treatment efficacy with the minimization of adverse effects. This case emphasizes the possibility of fast onset of hepatotoxicity in organ transplant recipients receiving AT therapy, highlighting the significance of closely monitoring liver function, particularly during the initial few weeks of treatment.

In patients with suspected AT drug-induced liver injury, symptoms such as hypoglycemia may suggest significant liver dysfunction, necessitating early intervention and reassessment of the treatment regimen.
